# Barreras del personal de salud para el tamizaje de sífilis en mujeres embarazadas de la Red Los Andes, Bolivia

**DOI:** 10.26633/RPSP.2017.21

**Published:** 2017-04-21

**Authors:** Freddy Tinajeros, Lucila Rey Ares, Vanessa Elías, Ludovic Reveiz, Franz Sánchez, Martha Mejía, Rosalinda Hernández, Rita Revollo

**Affiliations:** 1 Consultor independiente Consultor independiente Bolivia Consultor independiente; 2 Instituto de Efectividad Clínica y Sanitaria Instituto de Efectividad Clínica y Sanitaria Buenos Aires Argentina Instituto de Efectividad Clínica y Sanitaria; 3 Organización Panamericana de la Salud Organización Panamericana de la Salud Washington D.C. Estados Unidos de América Organización Panamericana de la Salud; 4 Ministerio de Salud y Deportes Ministerio de Salud y Deportes La Paz Bolivia Ministerio de Salud y Deportes; 5 Organización Panamericana de la Salud Organización Panamericana de la Salud La Paz Bolivia Organización Panamericana de la Salud

**Keywords:** Tamizaje masivo, sífilis, atención prenatal, embarazo, Bolivia, Mass screening, syphilis, prenatal care, pregnancy, Bolivia

## Abstract

**Objetivo.:**

Identificar barreras del personal de salud por las cuales las embarazadas que asisten al control prenatal no se realizan el tamizaje de sífilis (Red de Salud Los Andes, Bolivia).

**Métodos.:**

Se realizaron 46 entrevistas semiestructuradas a proveedores de salud y se analizaron los registros de 249 expedientes clínicos de embarazadas de ocho establecimientos públicos de salud de la Red Los Andes.

**Resultados.:**

Entre las barreras del personal de salud para el tamizaje de sífilis en embarazadas se identificaron el tiempo insuficiente del personal para sensibilizar sobre el beneficio del tamizaje de sífilis, algunos mencionaron que las pruebas de sífilis se deberían hacer solo en centros donde atienden partos y tienen laboratorio, la poca comunicación entre el personal de la consulta médica y laboratorio, así como también problemas de abastecimiento de suministros y reactivos. En la revisión de expedientes clínicos se observó que 55,4% contaba con los resultados de laboratorio de sífilis en sus expedientes y solo 37,4% de historias clínicas perinatales contaba con registro de resultados de laboratorios. A través de las entrevistas, se pudo observar que los proveedores perciben que el tamizaje de sífilis se realiza al 100% de las embarazadas que asisten al control prenatal.

**Conclusión.:**

El tamizaje para sífilis no se está realizando según lo establecido en la estrategia de país para la eliminación de la sífilis congénita, y no llega a más de la mitad de embarazadas en control prenatal con registros en las historias clínicas perinatales. Esto no es percibido por los profesionales de la salud y puede transformarse en una barrera para el tamizaje de sífilis en mujeres embarazadas.

## Abstract (en)

**Objective.:**

Identify health-worker barriers that keep pregnant women who receive prenatal care from being screened for syphilis (Los Andes Health Network, Bolivia).

**Methods.:**

Semi-structured interviews were done with 46 health care providers and 249 clinical health records of pregnant women were analyzed in eight public health facilities in the Los Andes network.

**Results.:**

Health-worker barriers to syphilis screening in pregnant women included lack of time by personnel to raise awareness of the benefit of syphilis screening; some mentioned that syphilis tests should only be done in facilities that attend deliveries and have a laboratory; lack of communication between clinicians and laboratory personnel; and problems with provision of supplies and reagents. The clinical record review found that only 55.4% contained syphilis laboratory results and only 37.4% of perinatal clinical histories had records of laboratory results. The interviews found that providers believe that syphilis screening is done in 100% of pregnant women receiving prenatal care.

**Conclusion.:**

Syphilis screening is not being done according to Bolivia’s strategy for the elimination of congenital syphilis, and is not done on more than half of pregnant women in prenatal care with perinatal clinical history records. This is not perceived by health professionals and can become a barrier to syphilis screening in pregnant women.

La sífilis constituye un problema de salud pública mundial, con un número estimado de 12 millones de personas infectadas y alrededor de 1,39-2,0 millones de gestantes cada año (1, 2). La infección suele ser asintomática en el embarazo y puede ocasionar mortinatos, muerte perinatal o infecciones neonatales graves. Sin embargo, opciones simples y rentables de detección y tratamiento durante el embarazo pueden eliminar la mayoría de estas complicaciones (3). En 2007, la Organización Mundial de la Salud (OMS) publicó la “Guía de Eliminación Mundial de la Sífilis Congénita: fundamentos y estrategia para la acción”. Esta estrategia busca aumentar el acceso global a las pruebas de sífilis y al tratamiento de las embarazadas. Para 2014, más de 40 países estaban haciendo la prueba para sífilis al 95% de mujeres embarazadas en el control prenatal (4). No obstante, aunque se han hecho progresos, muchos países aún deben priorizar la prevención y el tratamiento de la transmisión de vertical de la sífilis. En 2012, la sífilis afectó a 360 000 embarazos, con consecuencias como muerte fetal, muerte neonatal, prematuridad y recién nacidos infectados (5).

En Bolivia, según el estudio del Population Council realizado en las cuatro maternidades más grandes del país (Santa Cruz, La Paz, Cochabamba y El Alto) y en 37 comunidades rurales, se encontraron prevalencias de 3,5% a 6,7% en zonas urbanas y entre 1% y 15% en zonas rurales (6). Esta situación evidenció que el tamizaje adecuado de sífilis en embarazadas es una prioridad y que el tratamiento oportuno y adecuado previene la sífilis congénita (7) en casi el 100% de los casos (8).

El Alto es una ciudad ubicada en el departamento de La Paz, situada al oeste de Bolivia en la meseta del altiplano, a una altura de 4 000 m sobre el nivel del mar. En el año 2015 tenía 925 064 habitantes. Cuenta con 50 establecimientos públicos de salud de primer nivel de complejidad, tres hospitales de segundo nivel y un hospital de tercer nivel, todos distribuidos en cinco redes de salud. La Red de Salud Los Andes está conformada por ocho establecimientos públicos de primer nivel, un hospital de segundo nivel y un hospital de tercer nivel. Esta red de salud tiene una cobertura de 222 724 habitantes. El control prenatal de las embarazadas incluye el tamizaje de sífilis a través del Seguro Integral de Salud (SIS) en centros de primer nivel y se dispone de una guía para este fin.

En la Red de Salud Los Andes (2013) se estimaron 6 381 embarazos al año, se atendieron 5 642 partos institucionales y 4 882 gestantes con controles prenatales (3 118 antes del quinto mes y 1 764 después del quinto mes), lo que permite inferir que existen gestantes que llegan directamente al parto sin previo control prenatal (13,5%) y con un gran riesgo para la salud de las embarazadas: si padecen sífilis no se diagnostica a tiempo ni se puede prevenir la transmisión vertical.

Existen dificultades en el tamizaje de sífilis en el control prenatal, es por esta razón que existe una brecha entre el total de embarazadas que asisten al control prenatal y el total de las que se realizan el tamizaje para sífilis. Por otro lado, la notificación de casos de sífilis materna aun muestra dificultades en algunos centros de salud. El presente estudio pretende entender las barreras existentes para el tamizaje de sífilis en embarazadas.

## MÉTODOS

La investigación forma parte de la nueva iniciativa “Mejoras en la ejecución de programas a través de investigaciones acerca de dicha ejecución integrada (iPIER)”, desarrollada por la Alianza para la Investigación en Políticas y Sistemas de Salud (AHPSR) en colaboración con la organización Panamericana de la Salud (OPS). El modelo iPIER coloca a los ejecutores de programas en el centro de una investigación con el objetivo de entender las fallas en los sistemas de salud que crean barreras a la implementación, y permite también identificar las soluciones a estas barreras. La investigación sobre la ejecución integrada en los procesos existentes apoya la efectividad de los programas y políticas de salud políticas de salud eficaces a través de la utilización de la investigación que se llevó a cabo como parte del proceso de implementación. Una descripción detallada de la aplicación de la metodología de investigación se incluye en el documento conceptual iPIER (9).

Se definen como barreras a las dificultades que enfrentan las personas para acceder a los servicios de salud (10) los factores que influyen pueden ser desde el tiempo, la distancia, los trámites o el número de veces que debe retornar, hasta las condiciones favorables creadas por el sistema de salud para recibir el servicio determinado. Según Donabedian, la accesibilidad constituye un “factor mediador” entre la capacidad de producir servicios y el consumo real de dichos servicios (11).

**FIGURA 1:**
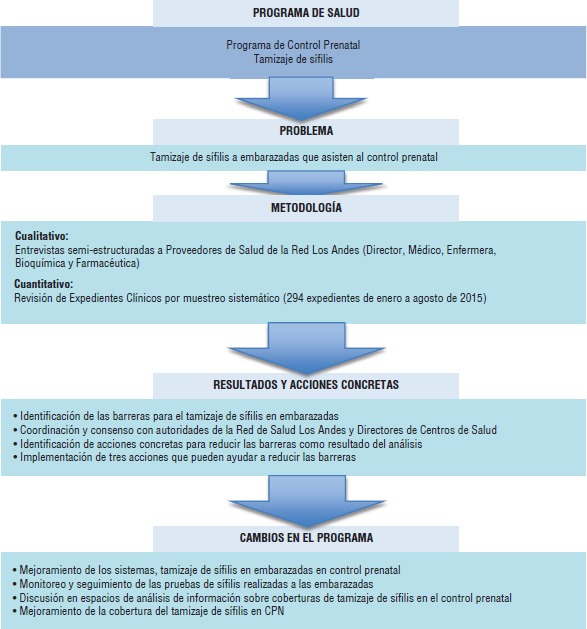
Flujograma del estudio barreras para el tamizaje de sífilis en embarazadas de la Red Los Andes, Bolivia, 2015.

El objetivo del presente estudio fue identificar las barreras del personal para el tamizaje de sífilis en mujeres embarazadas que asisten al control prenatal en la Red de Salud Los Andes y analizar los factores que limitan la disponibilidad de pruebas rápidas para sífilis. En la figura 1 se muestra de manera esquemática el problema que dio origen a este proyecto, así como las estrategias de implementación y cambios programáticos esperados.

El equipo de trabajo estuvo conformado por un investigador y consultor experto en infecciones de transmisión sexual (ITS) y virus de inmunodeficiencia humana (VIH), una investigadora de ITS y pediatra del Hospital Municipal Los Andes, el gerente de la Red de Salud Los Andes, tres profesionales que condujeron las entrevistas a los proveedores de salud y un equipo administrativo de la Fundación Colectivo Cabildeo. Esta combinación de competencias fortaleció la toma de decisiones en base a los resultados de la investigación.

El protocolo de investigación fue sometido para su aprobación al Comité de Revisión Ética de la OPS (PAHOERC por sus siglas en inglés).

El presente estudio utilizó métodos mixtos (cualitativo y cuantitativo). El componente cualitativo incluyó entrevistas semiestructuradas a proveedores de salud de la red Los Andes para conocer y entender las barreras para el tamizaje de sífilis (temas como suministros, solicitud de pruebas, tiempo, seguimiento y análisis de coberturas, entre otros). Las entrevistas fueron aplicadas por psicólogas y pedagogas con experiencia en estudios cualitativos, quienes recibieron entrenamiento en el protocolo. Los criterios de inclusión de los entrevistados fueron ser funcionario del Ministerio de Salud y que hubiera trabajado en un centro de la red Los Andes durante al menos seis meses (director, médico, enfermera, bioquímica y farmacéutica). Se planificó entrevistar a seis proveedores de cada centro de salud, hasta alcanzar saturación.

Se entrevistaron a 46 profesionales de salud (ocho directores, siete médicos, 16 enfermeras, siete bioquímicas y ocho farmacéuticas).

Las entrevistas fueron grabadas y transcritas en su totalidad.

El análisis de los datos cualitativos se realizó a través de una matriz de análisis, codificando las palabras clave que se repetían o se destacaban como relevantes en las entrevistas.

El componente cuantitativo se analizó a través de una revisión de los expedientes clínicos de control prenatal entre enero y agosto de 2015. Estos se seleccionaron a través de un muestreo aleatorio sistemático. El tamaño de muestra de 294 fue calculado por el *software* EpiDat^®^ tomando un universo de 2 940 embarazadas con control prenatal, asumiendo que alrededor de 70% de mujeres embarazadas se realizaron la prueba de sífilis en el 2013 (informe de cobertura Red Los Andes, 2013), con un intervalo de confianza de 95% (IC95%) y un margen de error de 5%.

Los datos extraídos de las historias clínicas de los ocho centros de salud de la red Los Andes fueron vaciados a una base de datos de Excel^®^ con listados desplegables. El análisis de datos se realizó utilizando Epi Info 7^®^. Se calculó el valor de *P* a partir de X^2^, con un IC95% para determinar si las diferencias encontradas fueron estadísticamente significativas.

## RESULTADOS

De los 294 expedientes clínicos, todos del primer control prenatal, 63% correspondieron a controles prenatales antes del quinto mes y 37% después del quinto mes.

Como resultado de las 46 entrevistas semiestructuradas aplicadas, se pudo observar que los proveedores de salud tienen la percepción que el tamizaje de sífilis se realiza al 100% de las embarazadas que asisten al control prenatal. Algunos de los entrevistados refieren que el tiempo para la atención no es suficiente. Indicaron que las pruebas de sífilis se deberían realizar solo en los centros que tienen laboratorio y donde se atiende partos y no en centros donde solo se realizan controles prenatales. También se mencionó que en las reuniones de los comités de análisis de información (CAI), estos temas se abordan de manera rápida y superficial, sin darle un seguimiento en detalle (cuadro 1). Uno de los factores que limita el suministro de pruebas rápidas son los desembolsos tardíos para la compra de las pruebas y sus insumos, lo cual genera como consecuencia el desabastecimiento.

Existe poca comunicación entre el personal de la consulta médica y el laboratorio respecto al tipo de pruebas de sífilis que se realizan, ya que estas pueden cambiar en el transcurso del tiempo. La mayoría de los entrevistados relacionan las barreras para el tamizaje de sífilis con la falta de información y el poco tiempo que disponen las embarazadas durante el control prenatal (cuadro 1).

El 100% de los entrevistados refiere que solicitan las pruebas de tamizaje de sífilis. Sin embargo en la revisión de los 294 expedientes clínicos, solo el 55,4% contaba con los resultados de laboratorio de sífilis en sus expedientes. Al analizar los centros por separado, se observaron los siguientes porcentajes de historias clínicas con registro: Alto Lima III 61,7%, Alto Lima IV 45,8%, Centro de Referencia Ambulatorio 61,1%, Germán Busch 62,1%, Huayna Potosí 67,3%, Puerto Mejillones 40,0%, Santa Rosa de Lima 13,3% y Villa Ingenio 51,1%, todos ellos pertenecientes a la Red de Salud Los Andes (cuadro 2).

**FIGURA 2:**
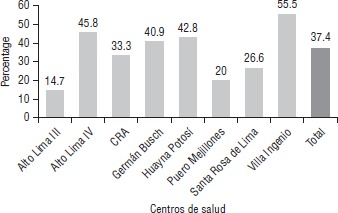
Porcentaje de historias clínicas perinatales con resultados de laboratorio de sífilis, Red de Salud Los Andes, Bolivia, 2015.

Al revisar las historias clínicas perinatales de las embarazadas, se muestra que el porcentaje de registro de resultados de laboratorio de sífilis es solo de 37,4% (figura 2), con el porcentaje más bajo registrado en Alto Lima III (14,7%) y el más alto en Villa Ingenio (55,5%).

## DISCUSIÓN

Tal como se pudo observar, el total de los entrevistados refiere que el tamizaje de sífilis se realiza a las embarazadas. Sin embargo, solo 55% de los expedientes clínicos revisados cuenta con resultados de laboratorio de sífilis y solo 37% de las historias clínicas perinatales tienen registro de los resultados de laboratorio de sífilis. Esta brecha existente podría ocasionar que una embarazada que recibió tamizaje de sífilis (12) en el control prenatal no cuente con esta información. Esto conlleva el riesgo de que si asiste al parto a otro centro de salud no reciba tratamiento adecuado o no se realice el seguimiento necesario al recién nacido, lo que implicaría repetir todas las pruebas ya realizadas. Por esta razón, es importante registrar los resultados de pruebas de sífilis en la historia clínica perinatal.

**CUADRO 1.** Barreras para el tamizaje de sífilis en embarazadas durante el control prenatal en centros de salud de la red Los Andes, El Alto, Bolivia, 2015

Categoría de análisis	Expresiones de las personas entrevistadas
Pruebas de Laboratorio en el control prenatal	“Como le dije, se hace a casi todas las mujeres embarazadas en su prenatal, se hace las pruebas en el laboratorio, en algún momento alguna por temor porque sacan la sangrecita una o dos veces no retornan de todas las mujeres que se hacen laboratorio”. (E5. Bioquímica) “De 100% de mujeres, el 98% regresa con resultado el 2%. En algunos casos he visto que no lo hacen, indican que no tienen tiempo, que tienen que atender a sus hijitos, ir a la escuela, no hay apoyo de la familia”. (E8. Médica) “Yo pienso que esta prioridad es más conveniente en lugares donde se atienden partos y tienen laboratorio, no en los centros que no lo hacemos porque existiría el doble pinchazo, pero se podría tener una prueba rápida tal vez como emergencia”. (E18. Bioquímica) “A veces tenemos desabastecimiento de pruebas rápidas, porque el desembolso es tardío y toma tiempo el cotizar y comprar y nos quedamos sin insumos y pruebas”. (E15. Bioquímica)
Barreras para el tamizaje de sífilis en el control prenatal	“Sería ideal poder contar con un poco más de tiempo. A veces, por la demanda o porque tenemos que atender diferentes situaciones al mismo tiempo, no podemos cubrir ese tiempo de orientación y sensibilización debida”. (E39. Médica) ”El tiempo en consulta es ingrato, no podemos dejar de estimar al menos 20 minutos en consulta con una mujer nueva”. (E12. Enfermera) “Hace meses no teníamos pruebas rápidas de sífilis y hasta que nos compraron tuvimos que pedir prestado de otro centro de salud, tratamos de solucionar”. (E35. Enfermera) “Así otra barrera es la poca información de la población, si se le insiste que lo cumplan en un tiempo requerido para que retornen, no cumplen”. (E42. Médico)
Análisis del tamizaje de sífilis en embarazadas en los CAI	“Ahora, en las tantas reuniones mensuales que tenemos se cuentan muy superficialmente los datos.” (E20. Directora de centro de salud)
Actitud de los proveedores de salud	“Pero el personal si está dispuesto para que mejore el tamizaje para la sífilis, es necesario”. (E23. Enfermera) “A partir de la voluntad de todos nosotros tenemos que lograr cobertura, tenemos tratar de hacer cumplir la ley porque nos trazamos una meta y si no encontramos es que no estamos buscando bien, el hecho por ejemplo que no tengamos sífilis diagnosticada hasta el momento… eso posiblemente se deba a que no estemos buscando bien.“(E36. Médica)

E, entrevistado/a; CAI; reuniones de análisis de información.

**CUADRO 2.** Cantidad total y porcentaje de expedientes clínicos con pruebas de laboratorio para sífilis. Red de Salud Los Andes, El Alto, Bolivia, 2015

Centro de salud	N	Porcentaje (%)	IC95%
Alto Lima III	34	61,7	43,5 – 77,8
Alto Lima IV	24	45,8	25,5 – 67,1
CRA	36	61,1	43,4 – 76,8
Germán Busch	66	62,1	49,3 – 73,7
Huayna Potosí	49	67,3	52,4 – 80,1
Puerto Mejillones	25	40,0	21,1 – 61,3
Santa Rosa de Lima	15	13,3	1,6 – 40,4
Villa Ingenio	45	51,1	35,7 – 66,3
Total	294	55,4	49,5 – 61,2

IC95%, intervalo de confianza de 95%; CRA, Centro de Referencia Ambulatorio.

Es probable, también, que las coberturas de tamizaje de sífilis sean mucho más altas. No obstante, una evidencia verificable fue la existencia del registro de resultados en la historia clínica perinatal, por lo cual también se deberá implementar un mejor seguimiento para que esta información esté disponible en los expedientes clínicos, y que los carnés perinatales cuenten con dichos resultados. El personal deberá planificar la solicitud de insumos y pruebas, considerando los tiempos de gestión del sistema de salud, para evitar el desabastecimiento.

Por otro lado, se han observado diferencias significativas (*P* < 0,05) entre los establecimientos de primer nivel de complejidad sin laboratorio (46,8%, IC95% 37,9-55,3) y los centros de segundo nivel con laboratorio (63,5%, IC95% 49,7-74,5). Esto permite plantear que es más probable que se realicen la prueba de sífilis en los establecimientos de segundo nivel que en los de primer nivel y que los resultados estén consignados en los expedientes clínicos, fundamentalmente en el carné perinatal.

Es necesario promover una mayor sensibilización en el personal de salud respecto a la sífilis, en vista de que la fase latente de la enfermedad puede durar muchos años (13). La percepción del personal de salud con respecto a que el tamizaje de sífilis se realiza a todas las embarazadas y que la brecha en cobertura es responsabilidad de la embarazada no ayuda a reducir las brechas. Se podría tener un mayor impacto al reconocer las debilidades y limitantes del sistema y, por lo tanto, también trabajar en la difusión de información y promoción del control prenatal en las mujeres embarazadas, sobre todo reforzando la importancia del tamizaje de sífilis y su efecto preventivo en la salud del recién nacido (14). El seguimiento y monitoreo deben ser implementados por el personal de salud en cada centro, de acuerdo a su realidad, con una discusión más profunda sobre el tema de las sífilis en las reuniones trimestrales de los CAI, con el liderazgo de las autoridades de salud y este debe ser un catalizador del sistema de salud, tal como sucedió en la experiencia de otros países (15).

El presente estudio permitió identificar las barreras y conocer la realidad del tamizaje de sífilis en la Red Los Andes, a partir del cual se implementaron acciones concretas para mejorarlo. Una de las limitaciones del estudio es que no se entrevistaron a embarazadas. Las entrevistas se realizaron solo al personal de salud en la Red Los Andes y no se pueden extrapolar los resultados a todos los centros de salud de El Alto. Aun así, es un punto de partida para generar recomendaciones y que a futuro se podría extender otras redes de El Alto y del país, incluyendo a embarazadas.

Si bien se han identificado algunas debilidades en el proceso del tamizaje de sífilis a embarazadas, sobre todo en el registro y archivo de los resultados en los expedientes clínicos, es necesario también destacar que la mayoría de los establecimientos de salud cuenta con fortalezas como la infraestructura, el personal capacitado y la actitud positiva para mejorar el tamizaje de sífilis en gestantes integrado al de VIH. Se debe recordar que la adherencia de la embarazada al sistema de salud depende en gran medida de la calidad de atención durante el control prenatal, parto y puerperio por profesionales de salud capacitados y sensibilizados (16).

Las acciones inmediatas para reducir las brechas fueron implementadas luego de conocer los resultados. Estas acciones fueron la inclusión de la columna de registro de pruebas de sífilis en los cuadernos de control prenatal y el seguimiento, monitoreo y supervisión del tamizaje por parte de los encargados en cada centro de salud (17). El análisis de este tema en los CAI, permitirán mejorar la cobertura de manera sostenible para que todas las embarazadas reciban una atención y seguimiento adecuados y sus bebés estén libres de sífilis congénita (18), con el lógico impacto económico (19). Según la Organización Mundial de la Salud (OMS), el fortalecimiento de los sistemas de salud ayudará a optimizar la salud a través de mejoras en una o más de las funciones, lo que resultará en mejores acceso, cobertura, calidad y eficiencia en el control de la sífilis en embarazadas (20).

Los resultados del estudio, permitieron sensibilizar a los proveedores de salud y a las autoridades de la Red de Salud Los Andes en la importancia del tamizaje de sífilis en embarazadas y el seguimiento a las coberturas. Se puede concluir que los resultados del estudio impactaron en forma positiva y ayudaron a reconocer la necesidad de un seguimiento y monitoreo más estrechos mediante la identificación e implementación de algunas acciones concretas para mejorar la cobertura del tamizaje de sífilis en embarazadas y su registro.

## Conclusión

El tamizaje para sífilis no se está realizando según lo establecido en la estrategia de país para la eliminación de la sífilis congénita, y no llega a más de la mitad de embarazadas en control prenatal, lo que no es percibido por los profesionales. Estos hallazgos podrían ser utilizados para diseñar intervenciones destinadas a lograr niveles de tamizaje óptimos, teniendo en cuenta las barreras y facilitadores que se identificaron en el estudio e implementando acciones de seguimiento a la realización de pruebas de sífilis y extender a otras redes de salud de El Alto, Bolivia.

## Conflictos de interés.

Ninguno declarado por los autores.

## Declaración.

Las opiniones expresadas en este manuscrito son responsabilidad del autory no reflejan necesariamentelos criterios ni la política de la RPSP/PAJPH y/o de la OPS.
